# Respiratory Syncytial Virus Infection Disrupts Monolayer Integrity and Function in Cystic Fibrosis Airway Cells

**DOI:** 10.3390/v5092260

**Published:** 2013-09-19

**Authors:** Kong Michele, Maeng Patrick, Hong Jeong, Szczesniak Rhonda, Sorscher Eric, Sullender Wayne, Clancy John Paul

**Affiliations:** 1 Departments of Pediatrics, University of Alabama at Birmingham, Birmingham, Alabama 35233, USA; 2 The Gregory Fleming James Cystic Fibrosis Research Center, University of Alabama at Birmingham, Birmingham, Alabama, 35233, USA; 3 Departments of Pulmonary Medicine, Cincinnati Children’s Hospital Medical Center, Cincinnati, Ohio 45229, USA; 4 Center for Global Health, Colorado School of Public Health, 13199 E Montview Blvd, Suite 310, A090 Aurora, CO 80045, USA

**Keywords:** Respiratory Syncytial Virus, bronchial epithelial cells, cystic fibrosis, F508del cystic fibrosis transmembrane conductance regulator (CFTR)

## Abstract

*Background*: Respiratory Syncytial Virus (RSV) infection is a common contributor to pulmonary symptoms in children with cystic fibrosis (CF). Here we examined RSV infection in immortalized bronchial epithelial cells (CFBE41o-) expressing wild-type (wt) or F508del cystic fibrosis transmembrane conductance regulator (CFTR), for monolayer integrity and RSV replication. *Methods*: CFBE41o- monolayers expressing wt or F508del CFTR were grown on permeable supports and inoculated with RSV A2 strain. Control experiments utilized UV-inactivated RSV and heat-killed RSV. Monolayer resistance and RSV production was monitored for up to six days post-infection. *Results*: Within 24 h, a progressive decrease in monolayer resistance was observed in RSV infected F508del CFBE41o- cells, while the monolayer integrity of RSV infected wt CFTR CFBE41o- cells remained stable. RSV replication was necessary to disrupt F508del CFBE41o- monolayers as UV-irradiated and heat killed RSV had no effect on monolayer integrity, with an earlier and much more pronounced peak in RSV titer noted in F508del relative to wt CFTR-expressing cells. RSV infection of wt CFBE41o- monolayers also resulted in blunting of CFTR response. *Conclusions*: These findings identify an enhanced sensitivity of CFBE41o- cells expressing F508del CFTR to RSV infection, replication and monolayer disruption independent of the cellular immune response, and provide a novel mechanism by which cystic fibrosis airway epithelia are susceptible to RSV-dependent injury.

## 1. Introduction

Cystic fibrosis (CF) is an autosomal recessive disorder, caused by mutations in the gene that encodes the cystic fibrosis transmembrane conductance regulator (CFTR) protein [1]. Deletion of phenylalanine at position 508 (F508del) is the most common disease causing mutation, resulting in protein misfolding and endoplasmic reticulum degradation of the CFTR protein [2]. In CF, CFTR dysfunction alters the airway surface liquid by depleting the periciliary liquid (PCL), ultimately resulting in increased mucous viscosity, decreased mucociliary clearance, and induction of airway inflammation [3]. Recent studies also demonstrate the importance of CFTR function in CF airway host defense, wound repair and airway remodeling [4,5], with CFTR dysfunction implicated in excessive lung inflammation, bacterial infection and colonization [6]. 

Epidemiologic studies have shown that pulmonary exacerbations caused by RSV disease, particularly in children with CF results in frequent acute outpatient visits and hospitalizations. In CF children, RSV can cause significant clinical manifestations [7,8,9], and may predispose to early respiratory colonization with CF-specific bacterial pathogens. Among infants with CF, RSV infection has been linked to earlier identification and colonization with *Pseudomonas aeruginosa* [10]. Yet, the mechanism by which RSV infection promotes severe symptoms in CF lungs and confers epithelial vulnerability to secondary bacterial infection is unknown. 

Previously Colasurdo *et al.* [11] used a CF mouse model to examine the relationship between the CFTR defect and lung disease caused by RSV infection. They demonstrated that CFTR deficient mice (CFTR^−/−^) had an exaggerated inflammatory response to the virus compared to control non-CF mice. Importantly CFTR^−/−^ mice exhibited an aberrant response to RSV infection, and had an impaired ability to clear the virus compared to non-CF mice. Despite previous reports suggesting the importance of mucociliary transport in viral-induced disease and pathophysiology [12], the expression and function of CFTR in RSV infected airway epithelia remains poorly described. Recently, others have shown that RSV infection downregulates ENaC expression and function, with blunting of CFTR activity *in vitro* [13,14,15]. The role of CFTR expression and activity in RSV infection and propagation, however, has not been assessed. Functional CFTR has also recently been shown to be a critical contributor to airway epithelial barrier function, but the role of CFTR in maintaining airway epithelial integrity following RSV infection remains unknown [16]. 

In the current study, we examined effects of RSV infection on human airway cell monolayers in the presence or absence of surface localized and functional CFTR. We hypothesized that CF cells would exhibit greater sensitivity to RSV infection, including enhanced monolayer disruption and increased viral load compared to matched wt CFTR expressing airway epithelial cells. 

## 2. Materials and Methods

### 2.1. Cell Line and Culture

Human bronchial epithelial cells (CFBE41o-) isolated from a CF patient (F508del/F508del) were originally immortalized and characterized by D. Gruenert *et al* [17,18]. The original parental CFBE41o- cells expressed endogenous F508del CFTR. Wild-type (wt) and F508del CFTR cDNA were then stably transduced into the cells using TranzVector™ (Tranzyme, Inc., Birmingham, AL) as previously described [19], and controlled for passage, cloning and phenotype prior to use. The TranzVector system produced stable lentiviral vector transduction with CFTR cDNA first cloned into the gene transfer component, under regulatory control of the human cytomegalovirus (CMV) promoter to generate the vector stock. CFTR expression was also coupled to the puromycin-N-acetyltransferase gene to allow for rapid selection of cells expressing CFTR in media containing puromycin. CFBE41o- cells were transduced at a multiplicity of infection (MOI) of one followed by puromycin selection. Puromycin-resistant cells were expanded to form a pool of stable CFTR expressors, which were subsequently selected to match wt and F508del CFTR expression [20]. 

For the experiments described below, cells were seeded on cell culture inserts (Transwell permeable supports, diameter 12 mm, 0.4 μm pores; Corning Life Sciences, Acton, MA, USA) in multi-well plates and kept submerged (under liquid-liquid conditions) until they were confluent. Subsequently, apical medium was removed from the upper compartment for 48 h prior to RSV infection to create an air-liquid interface. Cell monolayers were non-ciliated, had no mucous production and had resistance values of approximately 1,000 Ωcm^2^ prior to RSV infection. Cells were maintained in minimum essential media (MEM) supplemented with 10% fetal bovine serum (FBS), nonessential amino acids, and penicillin-streptomycin (PS, Invitrogen, Carlsbad, CA, USA) in a 5% CO_2_–95% air incubator at 37 °C. This airway epithelial cell line is well characterized and is known to polarize and preserve many features of the airway surface epithelium including CFTR channel gating activity, *Pseudomonas aeruginosa* biofilm formation and vectoral chloride transport [21,22]. This cell line was used because isogenic derivatives expressing wt CFTR and F508del CFTR are well characterized, thus allowing us to assess the direct contribution of wt *vs.* F508del CFTR to RSV infectivity and pathology (independent of donor to donor variability). 

#### 2.1.1. RSV Infection of Cell Model System

The RSV A2 strain used was obtained from American Type Culture Collection (ATCC, Manassas, VA, USA) and propagated in HEp-2 human nasopharyngeal carcinoma cells (CCL-23, ATCC, Manassas, VA, USA) and purified by centrifugation through a 35% sucrose cushion [23]. Wt and F508del CFTR CFBE41o- cell monolayers were infected with RSV at an MOI of 0.1. Cell media was collected at two-day intervals. RSV titers were determined by serial dilution and plaque assay in HEp2 cells [24] and expressed as Plaque Forming Unit (PFU)/milliliter. Control experiments utilized UV-inactivated RSV (eliminates viral infectivity without altering the conformation of viral proteins) and heat-killed RSV (eliminates viral infectivity and changes protein structures). For UV-inactivation of RSV, aliquots of RSV stocks were inactivated by exposure to 1,800 mJ of radiation in a Stratalinker UV cross-linker (Stratagene) and for heat-killed RSV, aliquots of viral stocks were boiled for 45 min [25].

#### 2.1.2. Transepithelial Resistance Measurement and Ussing Chamber Experiments

Transepithelial resistance (TER) provides a physical measure of the electrical resistance between airway epithelial cells and was monitored as a surrogate for monolayer integrity. TER was measured daily using a World Precision Instruments ohmmeter (WPI, Inc., Sarasota, FL, USA), and results are reported as mean±SEM Ωcm^2^. For experiments measuring CFTR dependent short circuit (I_SC_), CFBE41o- cells were cultured on 6 mm diameter permeable inserts and mounted into modified Ussing chambers (Jim’s Instruments, Iowa City, IA, USA) as previously described [20]. Monolayers were stimulated with adenosine (10 μM) and genistein (50 μM) to activate CFTR ion transport and blocked with CFTR inhibitor 172 (CFTR_INH_172, 10 μM, mucosal) as a further test of the CFTR dependent I_SC_.

### 2.2. Statistical analysis

A minimum of 12 monolayers for each condition constituted one experiment, and this was repeated three times with the results averaged. Descriptive statistics were computed for each study variable of interest, including means and standard error of the means (SEM). ANOVA was used to examine baseline differences in monolayer resistance. ANCOVA was applied to assess differences between groups across days with adjustment for baseline resistance. Linear mixed effects models were employed for group-specific changes in resistance parameters. An autoregressive lag-1 covariance structure with unequal group variances accounted for longitudinal measurements of resistance for each group. All statistical tests were performed using SigmaStat (Systat Software Inc., Chicago, IL, USA). Paired T-tests were used for analysis of short circuit current (I_SC_) and viral load, and a *p* value < 0.05 was used to assess statistical significance.

## 3. Results

### 3.1. Sensitivity of F508del CFTR Monolayers to RSV Infection

**Figure 1:**
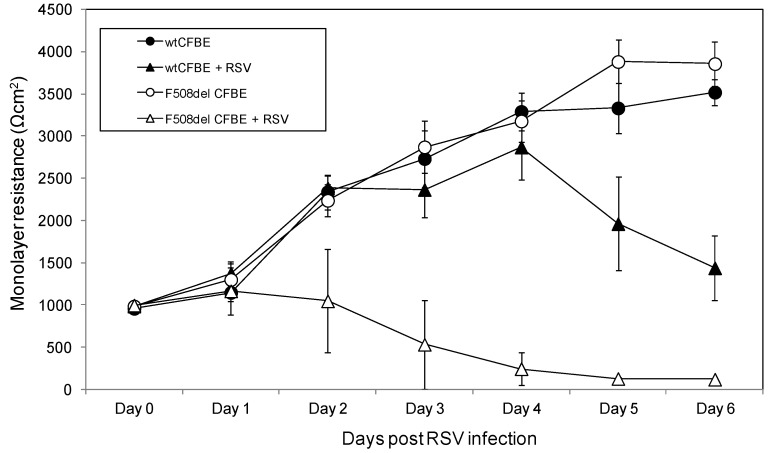
Resistance measurements across wt and F508del CFTR expressing CFBE41o- monolayers post RSV inoculation. Transepithelial resistance was measured daily (up to 6 days) in CFBE41o- cells transduced to overexpressed wild type (wt) CFTR or F508del CFTR. The white circles represent uninfected CFBE41o- cells expressing F508del CFTR while the black circles represent uninfected CFBE41o- cells expressing wt CFTR. The triangles represent CFBE41o- cells expressing wt CFTR (black) and F508del CFTR (white) infected with RSV A2 at a MOI of 0.1. Each time point represents the mean data obtained from 12 separate wells.

Transepithelial resistance was measured serially from Days 0 to 6 post RSV infection in wt and F508del CFTR expressing CFBE41o- cells. At baseline (prior to RSV infection), wt and F508del CFTR CFBE41o-monolayers had similar resistance values (971.67 ± 12.72 *vs.* 990.83 ± 9.88 mean ± SEM Ωcm^2^, respectively, Figure 1). In control (non-infected) cells, wt and F508del monolayers demonstrated steady and parallel increases in tissue resistance (+440.14 ± 37.19 and +515.24 ± 31.12 mean ± SEM Ωcm^2^ per day, respectively). In contrast, the resistance of F508del CFTR transduced cells infected with RSV rapidly decreased within 24 h and over several days post-infection (−236.62 Ωcm^2^ ± 27.96 mean ± SEM Ωcm^2^ per day; *p* < 0.0001). RSV infected wt CFTR monolayers exhibited daily increases in monolayer resistance (+447.17 ± 59.80 mean ± SEM Ωcm^2^; *p* < 0.0001) for 4 days, followed by a decrease for the remainder of the experiment (−523±70 mean ± SEM Ωcm^2^; ^∗^ *p* < 0.0001). Despite the declining resistance from days 4–6 in the RSV infected wt CFTR cells, monolayer resistance remains above the pre-RSV infection values at all time points. Resistance measurements in RSV-infected F508del CFBE41o- monolayers were significantly lower compared to RSV-infected wt CFBE41o- cells for days 2–6 (^†^ *p* < 0.0001 for all time points).

### 3.2. Live RSV Is Necessary for Disruption of Monolayer Integrity in F508del CFTR Monolayers

**Figure 2:**
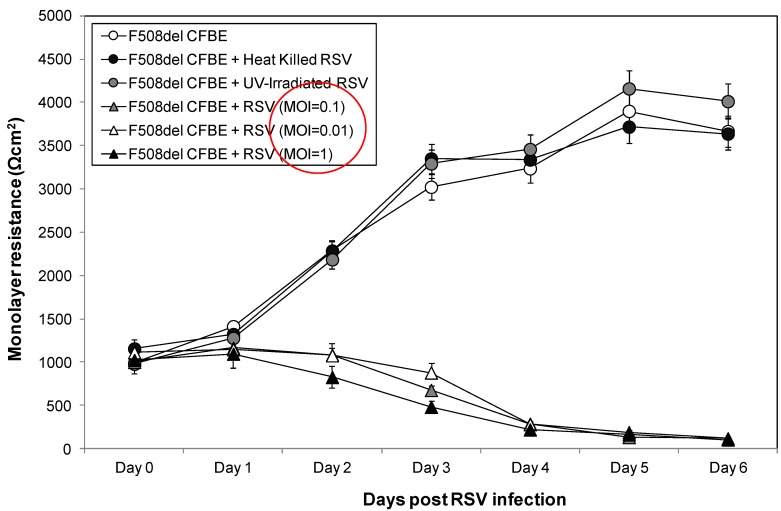
Resistance measurement across F508del CFTR expressing CFBE41o- cells post inoculation with live and killed RSV. Transepithelial measurements were obtained in uninfected CFBE41o- cells expressing F508del CFTR (white circles), as well as F508del CFTR cells infected with UV-irradiated RSV (grey circles), heat-killed RSV (black circles) and live RSV (MOI = 0.01 white triangles, MOI = 0.1 grey triangles and MOI = 1 black triangles). Monolayer resistance was measured daily from time of infection through Day 6 post-RSV.

CFBE41o- cells expressing F508del CFTR infected with UV-irradiated RSV and heat-killed RSV demonstrated similar monolayer resistance pattern as control uninfected F508del CFTR cell, with increasing resistance measurements over the course of the experiment (Day 0–6 post RSV infection, Figure 2). Our findings that treated RSV (with UV-irradiation and heat) had no effect on monolayer resistance over time compared with untreated control cells, indicated that RSV infection (and not exposure) was necessary for monolayer disruption. F508del CFTR monolayers were also exquisitely sensitive to RSV infection across three log doses of MOI (0.01, 0.1 and 1), with early rapid decline and low resistance measurements within 48 h. Monolayer resistance in the live RSV A2 infected F508del cells remained significantly decreased through Day 6 post-RSV infection for all three RSV infection titers relative to uninfected and both killed virus conditions (*p* < 0.0001 for all MOI).

### 3.3. Live RSV Infection Blunts CFTR Response in wt CFBE41o- Cells

**Figure 3:**
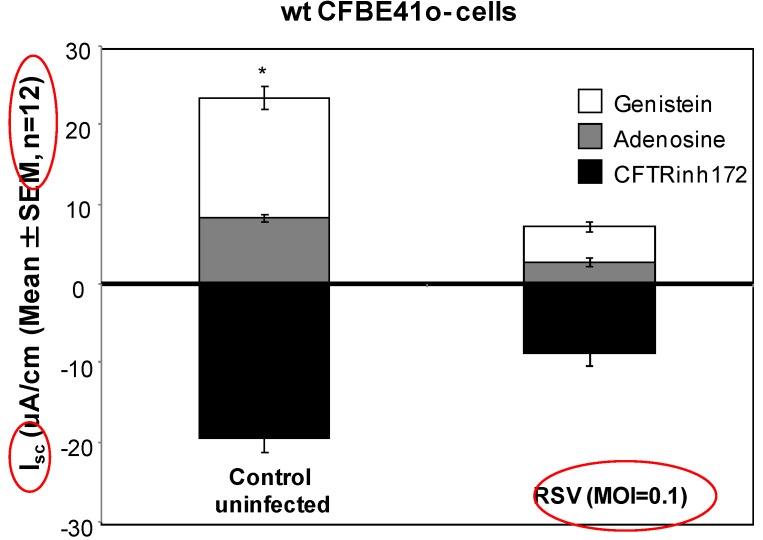
CFTR current measurements in control uninfected and RSV infected wt CFBE41o- cells. To measure CFTR dependent short circuit (I_SC_), CFBE41o- cells were cultured on 6 mm diameter permeable inserts and mounted into modified Ussing chambers. Cell monolayers were stimulated with adenosine (10 μM) and genistein (50 μM) to activate CFTR ion transport and blocked with CFTR_INH_172, (10 μM, mucosal) to confirm the CFTR dependence of I_SC_ changes.

We next examined the effects of RSV infection on CFTR function in polarized wt CFTR-transduced cells. We focused on CFTR-dependent chloride transport, as CFBE41o- cells transduced with wt or F508del CFTR demonstrate minimal ENaC expression and function [20]. Although wt CFBE41o- cells retained CFTR currents over four days post- RSV infection, the CFTR ion transport response to two activators [adenosine and genistein; −8.79 μA/cm^2^ (±2.91) in RSV-infected cells] was significantly blunted compared to control uninfected wt CFBE41o- cells [–19.39 μA/cm^2^ (±3.40) Figure 3, *p* < 0.05]. CFTR dependent I_SC_ was further confirmed by blockade with CFTR_INH_172 in both conditions.

### 3.4. Robust RSV Replication in F508del CFTR Monolayers

**Figure 4:**
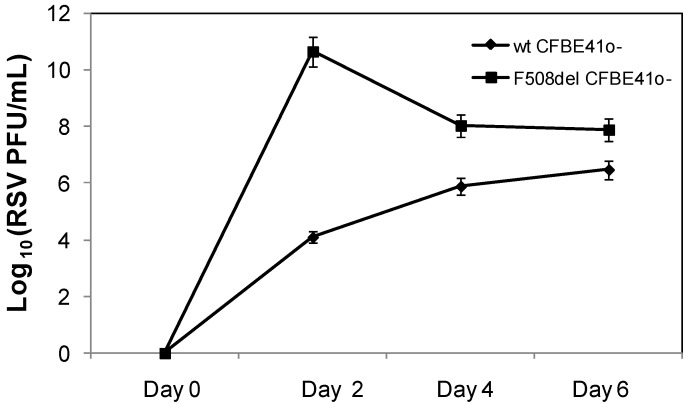
RSV titer in media of F508del and wt CFBE41o- cells with time. CFBE41o- cells transduced to overexpressed F508del (black square) or wt CFTR (black diamond) were inoculated with RSV (MOI = 0.1) at time point 0. Media from both cell lines were collected from the apical compartment at Days 2, 4 and 6 post RSV infection, and used to determine changes in viral load over time.

We next examined whether RSV replication was enhanced in F508del CFTR expressing cells relative to wt CFTR controls, and whether this correlated with the timeline for monolayer disruption (Figure 1 and 2). RSV replication was increased in F508del CFTR expressing cells relative to wt CFTR controls. This was associated with markedly elevated RSV titers within 48 h of infection in F508del CFTR cells relative to wt CFTR controls (Figure 4 and Table 1). In wt CFTR CFBE41o- cells, the RSV titer increased steadily from days 2–6 (Day 2: 1.3 ± 0.4 × 10^4^ PFU/mL, Day 4: 8 ± 0.7 × 10^5^ PFU/mL and Day 6: 3.1±0.5 × 10^6^ PFU/mL, *p* < 0.006 across the three time points). In contrast, F508del CFTR cells had the highest RSV titer on day 2 (4.6 ± 0.2 × 10^10^ PFU/mL), followed by a decrease in titer on days 4 and 6 post infection (Day 4: 1.1 ± 0.4 × 10^8^ and Day 6: 8.2 ± 1.1 × 10^7^ PFU/mL, respectively, *p* < 0.007 across all time points). Media from F508del CFTR monolayers had 3.5 × 10^6^ fold higher RSV titers relative to infected wt CFTR cells (*p* = 0.000009) on day 2 post infection. On day 4 and 6 post infection, measured RSV titers in F508del CFTR cells were 142-fold and 25-fold higher compared to viral load measured in wt CFTR monolayers (p = 0.000005 and 0.0002, respectively).

## 4. Discussion

In this study, we examined the influence of functional CFTR at the plasma membrane on RSV pathology in a widely utilized model of CF human airway epithelium. The use of matched wt and F508del CFTR airway cells allowed evaluation of epithelial behavior in a fashion independent of the cellular immune response, thus allowing us to focus on inherent epithelial responses. Emerging evidence supports a role for CFTR during airway epithelial wound repair, airway remodeling post injury, and monolayer barrier integrity as critical components in airway host defense [4,5]. In the present study, disruption of monolayer integrity was dramatically enhanced in CF airway epithelial cells (expressing F508del CFTR) relative to wt CFTR controls. These differences were manifest within 48 h of infection and progressed for the entire period of observation. The effects on monolayer integrity required viral replication, as UV-irradiated and heat killed RSV did not induce a decrease in transepithelial resistance. In a recent study by le Simple *et al.* [16], defective CFTR trafficking and tyrosine phosphorylation was implicated in modulation of CF airway epithelial barrier function via the regulation of paracellular permeability that was independent of CFTR-mediated chloride conductance. Singh *et al.* [26] reported a significant disruption in a non-CF epithelial membrane barrier secondary to RSV infection. The authors proposed that the decrease in transepithelial resistance indicated changes in paracellular permeability mediated by cellular cytoskeletal rearrangements. Our data indicate that human airway epithelial cells can be used to demonstrate CFTR-dependent RSV sensitivity, and implicate primary airway epithelial vulnerability to RSV infection. Although the decrease in transepithelial resistance did not appear to be clearly dependent on RSV concentration, our data suggest that F508del CFTR monolayers were exquisitely sensitive to RSV infection across several MOI (0.01 to 1), with monolayer disruption evident within 48 h of viral inoculation. It is possible that a higher MOI is required to demonstrate MOI dose-dependent effects, as suggested by a study in which MOI-dependent effects of RSV on a non-CF cell line were seen at an RSV MOI of 5 PFU/mL [26]. We hypothesize that CF airway epithelial cells are much more permissive to RSV replication, potentially contributing to the accelerated epithelial damage observed *in vivo*. 

Published results examining RSV infection in CF airway cells support the notion that the cystic fibrosis epithelium is uniquely susceptible to viral pathology. RSV infection has been linked to colonization with *Pseudomonas aeruginosa* [10,27] by facilitating bacterial adherence to the CF respiratory epithelium [28]. RSV has also been shown to up-regulate airway surface ATPase activity, causing depletion of the PCL and disruption mucociliary clearance [29]. The finding that RSV infection leads to a pronounced decrease in CFTR function at the cell surface (Figure 3) merits further investigative studies using primary human airway cells as depressed CFTR function identifies further disruption of normal airway surface liquid and PCL homeostasis, which could predispose to increased mucus viscosity, defective mucous clearance and exacerbation of secondary bacterial infection in non-CF airways *in vivo*. 

In the present study, RSV load and viral replication were significantly higher in CFBE41o- cells overexpressing F508del CFTR compared to those overexpressing wt CFTR (Figure 4). Peak viral yield was achieved within 48 h of infection in the F508del CFTR transduced cells, and decreased over time. Previous work with well-differentiated human airway epithelium cells found prolonged infection with RSV was associated with a decrease in the number of infected cells after peaking at 2–3 days. A reduction in the number of infected cells might correspond to a decrease in the production of infectious virus [30]. In contrast, viral titer increased steadily over six days in the wt CFTR condition, but remained below that of the F508del CFTR cells for the entire duration of our studies. A recent report by Vareille *et al*. [31] demonstrated increased susceptibility of the CF airway epithelium to rhinovirus infection that was associated with impaired antiviral early innate response. Similarly, Zheng *et al*. [32] reported increased human parainfluenza 3 replication in CF airway epithelial cell from lung explants secondary to lack of nitric oxide synthase 2 (NOS2) and 2’, 5’ oligoadenylate synthetase (OAS) induction in response to the virus. Furthermore, CF epithelial cells have been shown to exhibit increased viral replication compared to non-CF cells when exposed to human rhinovirus [33]. Additionally, RSV infection in a CF murine model demonstrated that CF mice had a defect in RSV clearance, with higher RSV burden compared to non-CF mice post infection, which was associated with increased lung inflammation and airway hyperresponsiveness [11]. In the present study, our data suggest that bronchial epithelial cells overexpressing F508del CFTR were inherently more sensitive to RSV replication. We postulate that this is in part secondary to the decreased monolayer integrity seen in RSV infected F508del *vs*. wt CFTR cells. Others have shown that increases in paracellular permeability of CF airway epithelia may be responsible for entry of bacterial toxins into the submucosa, resulting in increased susceptibility to bacterial infection [34].

To our knowledge, this is the first study that has examined the direct effect of RSV infection on the integrity of human airway epithelial cells expressing wt and F508del CFTR. The CFBE41o- cell line is a commonly used model for evaluating the consequences of F508del CFTR expression. These cells lack significant ENaC expression, allowing us to focus on CFTR-specific defects [25]. RSV infection has been shown to reduce ENaC activity *in vitro* and *in vivo*, including reduced expression of the gamma ENaC subunit, and downregulation of ENaC activity via inducible nitric oxide synthetase and P_2Y_ receptor signaling [13,14,15]. Here we demonstrate that RSV caused an early and pronounced loss of monolayer integrity independent of ENaC in cells expressing non-functional CFTR, and that the reduction in resistance was associated with higher RSV burden (Figure 4, Table 1). A limitation of this study includes the use of immortalized human airway cell lines. Although use of this *in vitro* model allowed for direct comparison between wt and F508del CFTR cells inoculated with RSV, future studies will require extension to primary human CF and non-CF airway epithelial cells.

**Table 1:** Increased RSV replication in F508del CFBE41o- cells. Media from F508del CFTR monolayers demonstrate higher RSV titers at all time points tested. In wt CFBE41o- cells, increasing RSV titer is noted from days 2–6. In contrast, F508del CFTR cells have the highest RSV titer on day 2, followed by decreasing viral titers on days 4 and 6 post-infection.

Days post RSV Inoculation	RSV titer (PFU/mL)		
	wt CFBE41o- cells	F508del CFBE41o- cells	*p*-values
Day 2	1.3 ± 0.4 × 10^4^	4.6 ± 0.2 × 10^10^	*p* = 0.000009
Day 4	8.0 ± 0.7 × 10^5^	1.1 ± 0.4 × 10^8^	*p* = 0.000005
Day 6	3.1 ± 0.5 × 10^6^	8.2 ± 1.1 × 10^7^	*p* = 0.0002

## 5. Conclusion

Our findings demonstrate that CF airway epithelia are highly susceptible to RSV infection, replication and monolayer disruption, and that the absence of wt CFTR expression leaves human airway cells vulnerable to pronounced epithelial injury by RSV. Our results implicate a critical CFTR-dependent barrier function that is defective during RSV infection in CF airway cells, potentially contributing to more severe pulmonary manifestations, including secondary bacterial infection and persistence in CF lungs.
